# Cervical cancer prevention in Ghana

**DOI:** 10.4314/gmj.v56i3.1

**Published:** 2022-09

**Authors:** Kwadwo A Koram

**Affiliations:** Department of Epidemiology, Noguchi Memorial Institute for Medical Research, University of Ghana, Legon, Ghana kkoram@noguchi.ug.edu.gh

Cervical cancer is the second most common cancer and is responsible for nearly 10% of all cancer deaths among women in the country.^1^ It is estimated that about 3000 deaths occur annually, and more tragically, many of these deaths can be prevented with a well-instituted preventive programme. Cervical cancer has been linked with infection with some Human Papilloma Virus (HPV) species in more than 95% of cases. HPV is a family of more than 150 species, but few of them have been linked with cervical, vulval and vaginal cancer in women; penile cancer in men; and throat, mouth and anal cancer in both men and women – the so-called high-risk HPV types.^2^

Primary prevention of cervical cancer has recently been made possible with the development and licensing of the HPV vaccine, which can be given to pre-adolescent children. Unfortunately, this has come late for most women at risk of the disease, and more importantly, the vaccines are currently unavailable for routine use in the country. Identification and treatment of pre-cancerous cells of the cervix is, thus, the main preventive activity available to adults. However, Pap smear testing, colposcopy and biopsy of suspicious lesions have remained unattainable to most at-risk populations. Colposcopy has remained the preserve of highly skilled gynaecologists, who are very few across the country. This has meant that, when available, screening has been limited to a few fixed centres and has largely been opportunistic. Estimates are that less than 5% of women who need testing are tested annually^2^. This results in the late presentation of cervical cancer cases with attendant high morbidity and catastrophic health expenditures for affected individuals and families (even though treatment is supposed to be covered under the NHIS). However, a well-planned and expanded screening program must be institutionalised to impact the burden of disease. Currently, there is no organised nationwide screening program, unlike in advanced countries.

However, the development of newer screening methods and the advancement of digital technologies provide a unique confluence of opportunities that can be exploited to impact this problem. When implemented, a combination of VIA and HPV screening can be a game changer. Mobile colposcopy and digital imaging can bring expertise to remote areas as all that is needed is a smartphone and connectivity. Furthermore, this is what the Cervical Cancer Prevention and Training Centre (CCPTC) at the Catholic Hospital, Battor, has been doing for the past five years.

In this issue of the journal, Effah and colleagues report in two papers aspects of their work showing the feasibility of establishing an affordable screening programme in the country. One paper showed that it is possible to train nurses to provide screening for women in remote areas using mobile colposcopy.^3^ In the other paper, they present data to show how a screening programme can be established on the back of the Ghana Health Service structure – from the CHPS compound and linked to the district hospital – the Hub and Spokes model, as they call it.^4^

The critical infrastructure needed to mount such a programme may be available at most district hospitals – HPV testing can be done on the same platform as the GeneXpert equipment provided to more than 130 district hospitals through the TB control programme. Leveraging this infrastructure and working through the GHS structure, every district can and should be encouraged to establish a screening programme. The initial cost of training and equipment provision may be daunting, but the service should not be deterred as the benefit will far outweigh this initial cost.

When trained nurses and other health workers are yearning to be employed, there is a large area of unmet needs for improving the population's health and women in particular – deployment of this cadre of health workers to provide preventive services. The group in Battor have shown, conclusively, how nurses can be used to provide essential health prevention to rural women. Indeed, it brings to the fore the whole concept of providing a skilled workforce as part of the essential function of public health. The time has come to see the expansion of our public health programs beyond the expanded program on immunisation (EPI), essential as that may be, to include the provision of preventive activities for the NCDs that are more intractable to treat /manage once they take hold.

The WHO launched the Cervical Cancer Elimination Initiative in 2020 with the targets of vaccinating 90% of girls below 15 years, screening 70% of women with a high-performance test by 35years and again by 45years and treating 90% of those with pre-cancerous lesions (90-70-90) by 2030.^5^ In areas with very few doctors or specialists, the adoption of innovative methods such as those presented in this issue may go a long way in helping the country move towards elimination of this preventable disease.
